# Structure-based screening of chemical libraries to identify small molecules that are likely to bind with the SET and RING-associated (SRA) domain of Ubiquitin-like, PHD and Ring Finger-containing 1 (UHRF1)

**DOI:** 10.1186/s13104-020-05103-4

**Published:** 2020-05-24

**Authors:** Debasis Patnaik

**Affiliations:** Meliorate Inc., North Weymouth, MA 02191 USA

**Keywords:** Cancer, UHRF1, SRA domain, UHRF1 inhibitor, DNA methylation, DNMT1, Structure-based screening, Virtual screening

## Abstract

**Objectives:**

UHRF1 is a multi-domain protein that recognizes both histone and DNA modification marks on chromatin. UHRF1 is involved in various cellular processes that lead to tumorigenesis and thus attracted considerable attention as a potential anti-cancer drug target. The SRA domain is a unique to the UHRF family. SRA domain recognizes 5-methylcytosine in hemimethylated DNA and necessary for maintenance DNA methylation mediated by DNMT1. Small molecules capable of interacting with the SRA domain may reduce aberrant methylation levels by preventing the interaction of 5-methylcytosine with the SRA domain and thereby blocking substrate access to the catalytic center of DNMT1. The data were collected to identify and predict an initial set of small molecules that are expected to bind to the SRA domain.

**Data description:**

Nearly 2.4 million molecules from various chemical libraries were screened with the SRA domain of UHRF1 using Schrodinger’s Small Molecule Drug Discovery Suite. The data is available in the form of a methodology presentation, MS Excel files listing the top hits, and Maestro pose viewer files that provide visualization of how the identified ligands interact with the SRA domain.

## Objective

UHRF1 functions as an epigenomic controller and is involved in various cellular mechanisms that lead to tumorigenesis [1]. UHRF1 has been proven to increase the activity and specificity of DNMT1 [2]. The SRA domain of UHRF1 is a DNA-binding domain and recognizes 5-methylcytosine (5mC) in hemimethylated CpG dinucleotides [3–7]. Due to the 5mC binding epitope architecture, the SRA domain is a highly promising site for small molecules targeting [8]. The SRA domain of UHRF1 interacts directly with DNMT1 and thereby provides improved substrate (hemimethylated DNA) access to the catalytic center of DNMT1, leading to an increase of DNA methylation activity [9]. In vitro studies have shown that UHRF1 can cause a fivefold increase in DNMT1 activity, and the SRA domain on its own can lead to a 1.9-fold increase in the activity of DNMT1. The interaction between UHRF1 and DNMT1 causes a nearly two-fold increase in the preferential targeting of hemimethylated DNA by DNMT1 [2]. Significantly, the expression levels of UHRF1 were described to be 5- to 70-folds lesser than those observed for HDAC1 and DNMT1 in healthy tissues. Thus, any potential adverse effects that may result due to the inhibition of UHRF1 expression or function are expected to be reasonably manageable when compared with consequences that are caused by the direct inhibition of DNMT1 [10]. Thus, preventing the interaction between the SRA domain and hemimethylated DNA via small molecules is a viable strategy to prevent aberrant DNA methylation [2]. Additional information about targeting the SRA domain for anti-cancer drug development was published earlier [1].

## Data description

The identification of small molecules that are predicted to bind to the SRA domain of UHRF1 was performed via virtual screening using Schrodinger’s Small Molecule Drug Discovery Suite. The crystal structures of UHRF1 is available in the public domain. The structure of the SRA domain and its interaction with hemimethylated DNA has been published [3, 5, 6]. The small molecule libraries were screened using the SRA domain (PDB Id: 3DWH) [7]. The downloaded PDB structure was prepared using the protein preparation wizard, which confirmed structural correctness at the start of the screening work. The Asp469 residue, which forms a hydrogen bond with the methylcytosine [6], was chosen as the active site, and a primary grid was prepared 10 A^0^ from the Asp469 residue [1]. The other residues that were selected to define the grid were Tyr466 and Tyr478 that sandwich 5-methylcytosine, and also Thr479, that is known to play a crucial role in the preferential recognition of cytosine [6].

A personal computer with the i7-4700MQ quad-core processor and 32 GB memory was used for this work. The small molecule libraries in the SDF format were prepared with LigPrep, to generate precise 3D molecular models for virtual screening. Epik was utilized for the consistent estimation of pKa values and to return chemically functional structures. The compounds were subjected to a filter to eliminate reactive compounds and analyzed via QIKPROP for the reliable projection of the ADME properties of the small molecules. The structure-based screening was performed using Schrodinger’s virtual screening workflow, which involves sequentially running Glide HTVS, Glide SP, and Glide XP on the prepared compound libraries. The virtual screening workflow removed 90% of the compounds at each phase, thus permitting only the top 10% of the small molecules on to the next step [1].

Nearly 2.4 million small molecules were screened using the SDF files of compound libraries from ChemDiv (San Diego, CA) and Timtec (Newark, DE). The numbers mentioned in parenthesis is the number of small molecules of the library. TIMTEC’s libraries include the Actimol collection (127,937), HTS part I, and HTS part II (400,000 & 491,349). ChemDiv libraries that were screened were Discovery Chemistry 1, 2 and 3 (350,000, 350,000 and 277,772) and New Chemistry 1 and 2 (250,000 and 206,249). The focused libraries from ChemDiv that were screened include bromodomain (6114), cancer stem cells (19,956), 3D mimetics (9461), soluble diversity (9624), targeted diversity (46,817), and methyltransferase (11,647) libraries. The specific libraries were chosen to facilitate the identification of diverse drug-like molecules that are likely to interact with an anti-cancer drug target with a crucial role in epigenomic regulation.

The data is available in the form of Maestro pose viewer files that is output by Glide. Glide is a sophisticated numerical algorithm optimized for docking accuracy and database enrichment. The pose viewer file contains a set of selected entries in Maestro in which the first entry is the protein (SRA domain), and all the other entries are poses of the docked ligand. After entering into the Pose Viewing Mode, the ligand poses can be navigated. The output files thus provide information about the identified molecules and visualize the predicted interactions with the SRA domain of UHRF1 (Table 1).

**Table 1:** Overview of data files/data sets

Label	Name of data file/data set	File types (file extension)	Data repository and identifier (DOI or accession number)
Data file 1	Patnaik SRA domain methodology for Structure Based Screening	Microsoft (MS) PowerPoint Presentation (.pptx)	10.6084/m9.figshare.12086727 [11]
Data file 2	3DWH ChemDiv Bromodomain	MS Excel Worksheet (.xlsx)	10.6084/m9.figshare.12086727 [11]
Data file 3	3DWH ChemDiv CancerStemCell	MS Excel Worksheet (.xlsx)	10.6084/m9.figshare.12086727 [11]
Data file 4	3DWH ChemDiv HelicalMimetics	MS Excel Worksheet (.xlsx)	10.6084/m9.figshare.12086727 [11]
Data file 5	3DWH ChemDiv Methyltransferase	MS Excel Worksheet (.xlsx)	10.6084/m9.figshare.12086727 [11]
Data file 6	3DWH ChemDiv SolubleDiversity	MS Excel Worksheet (.xlsx)	10.6084/m9.figshare.12086727 [11]
Data file 7	3DWH ChemDiv TargetedDiversity	MS Excel Worksheet (.xlsx)	10.6084/m9.figshare.12086727 [11]
Data file 8	vsw_1 3DWH actimol FEb082015-XP_OUT_1	MS Excel Comma Separated Values File (.csv)	10.6084/m9.figshare.12086727 [11]
Data file 9	vsw_1 3DWH actimol FEb082015-XP_OUT_1_pv	Compressed Maestro file (.maegz)	10.6084/m9.figshare.12086727 [11]
Data file 10	vsw 3DWH Bromodomain-XP_OUT_1	MS Excel Comma Separated Values File (.csv)	10.6084/m9.figshare.12086727 [11]
Data file 11	vsw 3DWH Bromodomain-XP_OUT_1_pv	Compressed Maestro file (.maegz)	10.6084/m9.figshare.12086727 [11]
Data file 12	vsw3DWHBromodomain-XP_OUT_1	Text Document (.log)	10.6084/m9.figshare.12086727 [11]
Data file 13	vsw3DWHChemDivCancerStemCell-XP_OUT_1	MS Excel Comma Separated Values File (.csv)	10.6084/m9.figshare.12086727 [11]
Data file 14	vsw3DWHChemDivCancerStemCell-XP_OUT_1	Text Document (.log)	10.6084/m9.figshare.12086727 [11]
Data file 15	vsw3DWHChemDivCancerStemCell-XP_OUT_1_pv	Compressed Maestro file (.maegz)	10.6084/m9.figshare.12086727 [11]
Data file 16	vsw3DWHDiscChem03-XP_OUT_1	MS Excel Comma Separated Values File (.csv)	10.6084/m9.figshare.12086727 [11]
Data file 17	vsw_13DWHChemDivDISCCHEM01Feb102015-XP_OUT_1	Text Document (.log)	10.6084/m9.figshare.12086727 [11]
Data file 18	vsw_13DWHChemDivDISCCHEM01Feb102015-XP_OUT_1_pv	Compressed Maestro file (.maegz)	10.6084/m9.figshare.12086727 [11]
Data file 19	vsw_13DWHDiscChem02-XP_OUT_1	MS Excel Comma Separated Values File (.csv)	10.6084/m9.figshare.12086727 [11]
Data file 20	vsw_13DWHDiscChem02-XP_OUT_1	Text Document (.log)	10.6084/m9.figshare.12086727 [11]
Data file 21	vsw_13DWHDiscChem02-XP_OUT_1_pv	Compressed Maestro file (.maegz)	10.6084/m9.figshare.12086727 [11]
Data file 22	vsw3DWHDiscChem03-XP_OUT_1	MS Excel Comma Separated Values File (.csv)	10.6084/m9.figshare.12086727 [11]
Data file 23	vsw3DWHDiscChem03-XP_OUT_1	Text Document (.log)	10.6084/m9.figshare.12086727 [11]
Data file 24	vsw3DWHDiscChem03-XP_OUT_1_pv	Compressed Maestro file (.maegz)	10.6084/m9.figshare.12086727 [11]
Data file 25	vsw_13DWHChemDivHelMimetics-XP_OUT_1	MS Excel Comma Separated Values File (.csv)	10.6084/m9.figshare.12086727 [11]
Data file 26	vsw_13DWHChemDivHelMimetics-XP_OUT_1	Text Document (.log)	10.6084/m9.figshare.12086727 [11]
Data file 27	vsw_13DWHChemDivHelMimetics-XP_OUT_1_pv	Compressed Maestro file (.maegz)	10.6084/m9.figshare.12086727 [11]
Data file 28	vsw3DWHmethyltransferase-XP_OUT_1	MS Excel Comma Separated Values File (.csv)	10.6084/m9.figshare.12086727 [11]
Data file 29	vsw3DWHmethyltransferase-XP_OUT_1	Text Document (.log)	10.6084/m9.figshare.12086727 [11]
Data file 30	vsw3DWHmethyltransferase-XP_OUT_1_pv	Compressed Maestro file (.maegz)	10.6084/m9.figshare.12086727 [11]
Data file 31	vsw3DWHnewChem01-XP_OUT_1	MS Excel Comma Separated Values File (.csv)	10.6084/m9.figshare.12086727 [11]
Data file 32	vsw3DWHnewChem01-XP_OUT_1	Text Document (.log)	10.6084/m9.figshare.12086727 [11]
Data file 33	vsw3DWHnewChem01-XP_OUT_1_pv	Compressed Maestro file (.maegz)	10.6084/m9.figshare.12086727 [11]
Data file 34	vsw3DWHnewChem02-XP_OUT_1	MS Excel Comma Separated Values File (.csv)	10.6084/m9.figshare.12086727 [11]
Data file 35	vsw3DWHnewChem02-XP_OUT_1	Text Document (.log)	10.6084/m9.figshare.12086727 [11]
Data file 36	vsw3DWHnewChem02-XP_OUT_1_pv	Compressed Maestro file (.maegz)	10.6084/m9.figshare.12086727 [11]
Data file 37	vsw_1ChemDivSMART-XP_OUT_1	MS Excel Comma Separated Values File (.csv)	10.6084/m9.figshare.12086727 [11]
Data file 38	vsw_1ChemDivSMART-XP_OUT_1	Text Document (.log)	10.6084/m9.figshare.12086727 [11]
Data file 39	vsw_1ChemDivSMART-XP_OUT_1_pv	Compressed Maestro file (.maegz)	10.6084/m9.figshare.12086727 [11]
Data file 40	vsw3DWHSolDiv-XP_OUT_1	MS Excel Comma Separated Values File (.csv)	10.6084/m9.figshare.12086727 [11]
Data file 41	vsw3DWHSolDiv-XP_OUT_1	Text Document (.log)	10.6084/m9.figshare.12086727 [11]
Data file 42	vsw3DWHSolDiv-XP_OUT_1_pv	Compressed Maestro file (.maegz)	10.6084/m9.figshare.12086727 [11]
Data file 43	vsw3DWHTargDiver-XP_OUT_1	MS Excel Comma Separated Values File (.csv)	10.6084/m9.figshare.12086727 [11]
Data file 44	vsw3DWHTargDiver-XP_OUT_1	Text Document (.log)	10.6084/m9.figshare.12086727 [11]
Data file 45	vsw3DWHTargDiver-XP_OUT_1_pv	Compressed Maestro file (.maegz)	10.6084/m9.figshare.12086727 [11]

## Limitations

- The present investigation is limited to the selected small molecule libraries from ChemDiv and Timtec.
- The structure-based virtual screening was carried out using most of the default parameters of the Schrodinger’s Small Molecule Drug Discovery Suite.
- The small molecule hits that were identified in the present study only narrow down the number of compounds that needs to be evaluated initially in an in vitro assay.
- The small molecules identified in this study have not been evaluated in a biochemical or biophysical assay. Some of the identified small molecules may not show a binding response to the SRA domain of UHRF1 in a biochemical or biophysical assay. If a successful binding interaction is detected in an in vitro assay, the molecules need to be validated further in a series of biochemical, biophysical, and cell-based assays.

## Data Availability

The data described in this Data note can be freely and openly accessed on Figshare (10.6084/m9.figshare.12086727). See Table 1 for details and links to the dataset [11].

## Abbreviations

UHRF1: Ubiquitin-like containing PHD Ring Finger 1
SRA: SET and RING-associated domain
SET: Su(var)3-9, enhancer-of-zeste and Trithorax
RING: Really interesting new gene
DNMT1: DNA methyltransferase 1
HDAC1: Histone deacetylase 1
PDB: Protein Data Bank
